# Mathematical modelling of a single tethered aerostat using longitudinal stability derivatives

**DOI:** 10.1038/s41598-024-53851-1

**Published:** 2024-02-14

**Authors:** Anoop Sasidharan, Ratna Kishore Velamati, Akram Mohammad, Sabrina Benaissa

**Affiliations:** 1 Department of Electrical and Electronics Engineering, Amrita Vishwa Vidyapeetham, Amritapuri, India; 2 Department of Mechanical Engineering, Amrita School of Engineering, Amrita Vishwa Vidyapeetham, Coimbatore, India; 3 Department of Aerospace Engineering, King Abdulaziz University, Jeddah, Saudi Arabia; 4 Department of Sciences and Technology, Faculty of Technology, University of Batna 2, Batna, Algeria; 5 Laboratory LPEA, University of Batna 1, Batna, Algeria

**Keywords:** Tethered aerostat, Stability derivatives, Longitudinal dynamic model, CFD, Energy science and technology, Applied mathematics, Engineering

## Abstract

Lighter-than-air (LTA) aerial vehicles such as airships and aerostats can be found in various strategic and commercial applications, primarily due to their capability to hover and stealth. The mathematical model of these vehicles helps in understanding their complex dynamics and designing and developing proper stabilisation systems for them. Stability derivatives have been used for developing mathematical models for heavier-than-air aerial vehicles since their introduction. This paper presents a methodology to develop a mathematical model of an aerostat based on stability derivatives. One of the major contributions of this study is the estimation of aerostat’s added mass terms expressed as longitudinal stability derivatives due to acceleration of the longitudinal motion variables. A longitudinally decoupled linear mathematical model of a single-tethered aerostat using stability derivatives is investigated in this study. A computational fluid dynamics (CFD)-based analysis of the 3D model of the vehicle is used to obtain the stability derivatives. The methodology presented considers the aerostat and tether models separately before coupling them to create the full model. The stability derivative analysis is carried out using ANSYS Fluent, and the coupled tethered aerostat model is investigated using MATLAB 2020. The negative pitch angle of the aerostat is caused by the selection of the pitching centre as the aerostat centre of volume instead of the tether confluence point. The tension force on the tether, which is proportional to the wind velocity, and aerostat velocity components are found to be stabilised within 200–400 s.

## Introduction

The mathematical modelling of aerostats is similar to that of aircraft in the sense that the dynamics involved in the operation of both vehicles are similar, with some exceptions. The significant difference in the dynamic model of a lighter-than-air (LTA) aerial vehicle from that of other aerial vehicles is the inclusion of the buoyancy effect, the added mass effect, and the tether dynamics.

The literature regarding the mathematical modelling of aerostats can be broadly classified based on the methods used for obtaining the model parameters as analytical methods, experimental methods, and numerical methods. It can be seen that the aerodynamic modelling of aerostats was presented first in a detailed manner by Munk and then followed by other contributors^1,2^. The majority of the literature on the aerodynamic model of aerostats is based on the work of Jones and DeLaurier^3^. Some papers provided analytical modelling of the aerodynamics of LTA vehicles, but the models heavily relied on experimental processes and were not applicable to all vehicles^4–7^.

As the impact of the aerodynamic effects on the aerial vehicle in the form of force and moment terms is not easily measured, mathematical derivative forms are required to represent these force and moment terms^8,9^. The aerodynamic stability derivatives have been adopted by the research community as one of the foundations for modelling the aerodynamic impacts in the mathematical equations of aerodynamic systems since their introduction by Bryan in 1911^10^. Aerodynamic models based on stability derivatives are regarded as an accurate method for mathematically modelling heavier-than-air aerial vehicles.

Flight experiments, wind tunnel testing, and numerical approaches were mostly used to estimate or extract the stability derivatives. A few of the critical works available in these directions are presented here. Jones reported the calculation of the aerodynamic stability derivatives of the LTA vehicles based on a semi-empirical procedure^3^. They have also analysed the TCOM 71 M aerostat’s longitudinal and lateral stability derivatives^11^. The model developed using the stability derivatives also validated the aerostat’s static and dynamic stability. Ignatyev demonstrated a methodology based on neural networks to predict the aircraft’s dynamic stability derivatives at high AoA^12^. In comparison to wind tunnel experimental data, they produced reasonable estimates for pitching moment derivatives. Asrar reported the methodology for extracting the stability derivatives of buoyant hybrid aerial vehicles using wind tunnels^13^. They compared the vehicle’s stability with and without wings. Despite the closeness to the real conditions of the experimental approaches, such as flight testing and wind tunnel experiments, they have several drawbacks, such as scaling inaccuracies, high cost, and blocking effects. Tao presented an approach based on system identification to obtain the derivatives of an LTA vehicle that is having swing oscillations^14^. Computational approaches for estimating the stability derivatives of LTA vehicles were also reported^15^.

Due to advancements in digital computing technology, numerical CFD solvers are suitable for handling high-performance computations^16–19^. As the stability derivative extraction of aerodynamic structures is very expensive in terms of computational power, the research community is utilising numerical solvers for extraction^20–22^. The stability derivatives were calculated using a forced oscillation of the body. According to the linear theory, all variables involved in the motion other than the oscillation parameter are neglected. To compute the corresponding dynamic derivative, a forced periodic and sustained oscillation is applied to the model’s respective degree of freedom (DoF). For example, the stability derivatives due to the pitching moment can be extracted by applying such an oscillation on the pitch axis^9^. The process of extraction involves the application of Fourier theory to the measured moment response of the model. Such an approach can be found in the work by Wang, in which some of the derivatives of an airship were derived^23^. They reported the oscillation along the pitch and heave motion axes of the airship, along with some findings about the effect of tail fins on the vehicle’s stability. An investigation into the stability derivatives of a heavier-than-air vehicle is reported by Ronch. They have considered the aerodynamic impact on the aircraft flight dynamics at transonic speeds and considerably large angles of attack^24,25^.

Although there are reports of the investigation of stability derivatives of LTA vehicles, mathematical models using these derivatives have not been reported. The major contribution of this paper is to present the mathematical model of a tethered aerostat based on the longitudinal stability derivatives extracted using CFD analysis. The added mass terms, which are generally complex parameters to obtain, estimated using the presented methodology is also a contribution of the current study. The rest of the paper is organised as follows: Section “Mathematical modelling of the tethered aerostat” presents the mathematical modelling of the tether and the aerostat. The methodology adopted for extracting stability derivatives of the aerostat involved in the aerodynamic part of the model is also explained in this section. Section “Results and discussion” analyses the responses of the developed model. It includes the validation study results conducted as part of the investigation and the tethered aerostat model responses for different wind conditions. Section “Conclusion” concludes the paper with major findings and future directions.

## Mathematical modelling of the tethered aerostat

### Tether model

**Figure 1:**
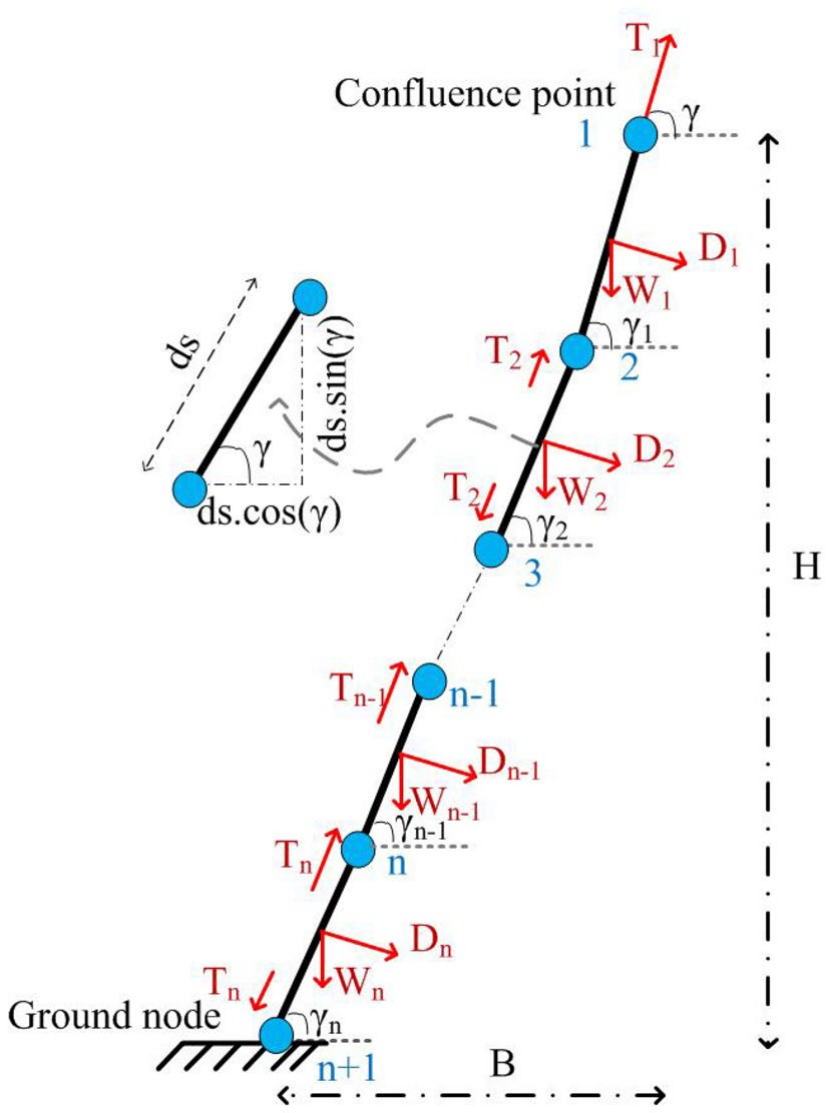
Two-dimensional discretized tether model.

The tether for the aerostat is modelled as a series of segments with equal lengths^26^. Figure 1 shows the discretized tether representation with the forces marked. The length of each segment is $d_s~=~S/n$, where *S* is the total length of the tether and *n* is the number of segments. For *n* tether segments, there will be $n+1$ nodes. The forces acting on the tether are tension (*T*), gravitational force (*W*), and drag force (*D*). These forces are distributed among the tether segments, as shown in the figure. The altitude of operation is *H*, i.e., the top node will be at this altitude.

The model of the tether can be obtained by considering the force balance along the horizontal and vertical directions. Forces along the horizontal and vertical directions can be expressed as in Eqs. (1) and (2), respectively.

1 $$\begin{aligned}{} & {} T_i\cos {\gamma _i}+D_i\sin {\gamma _i}=T_{i+1}\cos {\gamma _{i+1}} \end{aligned}$$

2 $$\begin{aligned}{} & {} T_i\sin {\gamma _i}=D_i\cos {\gamma _i}+W_i+T_{i+1}\sin {\gamma _{i+1}} \end{aligned}$$

where $T_i$ and $T_{i+1}$ are the tensions of the *i*th and ${i+1}$th nodes of the tether; $\gamma _i$ and $\gamma _{i+1}$ are the tether angles with the horizontal for the *i*th and $i+1$th nodes of the tether; and $D_i$ and $W_i$ are the normal drag component and weight of the *i*th segment, respectively.

The expression for the angle between the tether segment and the horizontal in terms of the previous node can be obtained as in Eq. (3), and the expression for tension can be obtained as in Eq. (4).

3 $$\begin{aligned} \gamma _{i+1}= & {} \tan ^{-1}\left( \frac{T_i\sin {\gamma _i}-D_i\cos {\gamma _i}+W_i}{T_i\cos {\gamma _i}+D_i\sin {\gamma _i}}\right) \end{aligned}$$

4 $$\begin{aligned} T_{i+1}= & {} \frac{T_i\cos {\gamma _i}+D_i\sin {\gamma _i}}{9}\cos {\gamma _{i+1}} \end{aligned}$$

The components of the tether tension for the model can be obtained by resolving the tension into their respective components.

### Aerostat model

**Figure 2:**
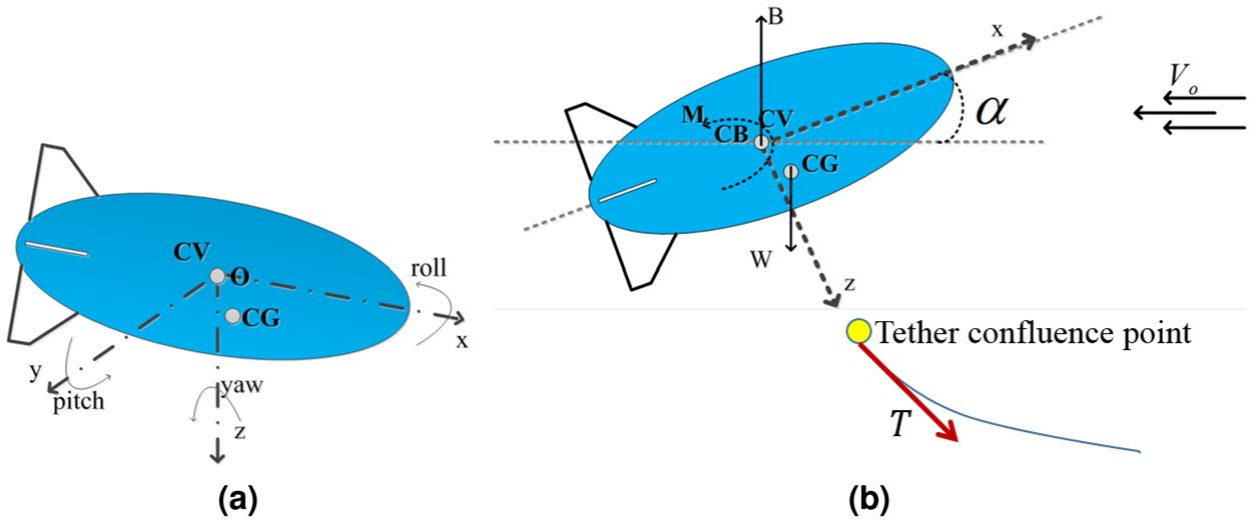
(**a**) Orientation of the aerostat with respect to the body axes; and (**b**) steady rectilinear condition of the aerostat.

The longitudinal model of the aerostat is developed based on two reference frames, namely the body-fixed reference frame and the inertial reference frame^27^. Figure 2a shows the body fixed frame (*Oxyz*) and its orientation. The origin of the body frame is at the centre of volume. Axis *Ox* points towards the leading edge, *Oy* points towards the starboard side, and *Oz* points downwards.

A steady rectilinear operating condition is considered for the model, with $V_0$ as the free stream wind velocity and $\Theta _e$ as the aerostat attitude, as shown in Fig. 2b. The relative velocity of the aerostat with respect to the wind can be expressed as $V_0=\sqrt{(U_e+V_e+W_e )}$. For a perturbed condition, the total velocity can be expressed as,

5 $$\begin{aligned} \begin{aligned} \grave{u}&=u+U_e\\ \grave{v}&=v+V_e\\ \grave{w}&=w+W_e \end{aligned} \end{aligned}$$

where *u*, *v*, and *w* are the perturbed variables. Under steady conditions, these perturbed variables and their derivatives are zero.

The mathematical model of the aerostat consists of Newton’s second law of motion applied to each of the concerned DoFs as follows:

6 $$\begin{aligned} {\textbf {ma}}={\textbf {F}} \end{aligned}$$

where ${\textbf {m}}$ is the mass, ${\textbf {a}}$ is the acceleration, and ${\textbf {F}}$ is the disturbing force. For rotational DoFs, the mass, acceleration, and force terms become inertia, angular acceleration, and moment, respectively.

The aerostat’s longitudinal motion is taken into account in this work. It is assumed that the control surfaces of the LTA vehicle compensate for the lateral dynamics and motion of the vehicle. The presented longitudinally decoupled dynamic model includes the motion variables *u*, *w*, and *q*. Setting the lateral dynamic variables and the product terms in Eq. (6) as zero leads to the linearized longitudinally decoupled model of the aerostat as given in Eq. (7).

7 $$\begin{aligned} \begin{aligned}{}&m_x\dot{u}+\dot{q}(ma_z-\dot{\bar{D}}_{\dot{q}})=D_T+D_g+D_a\\&m_z\dot{w}-\dot{q}(ma_x+\dot{\bar{L}}_{\dot{q}})=L_T+L_g+L_a\\&J_y\dot{q}+\dot{u}(ma_z-\dot{\bar{M}}_{\dot{u}})-\dot{w}(ma_x+\dot{\bar{M}}_{\dot{w}})=M_T+M_g+M_a \end{aligned} \end{aligned}$$

where, the terms $M_a$, $D_a$, and $L_a$ represent the aerodynamic forces, $M_T$, $D_T$, and $L_T$ represent the tether force, and $M_g$, $D_g$, and $L_g$ represent the buoyancy force and gravitational force. *m* is the aerostat mass, and $m_x=m-\dot{\bar{D}}_{\dot{u}}$, $m_z=m-\dot{\bar{L}}_{\dot{w}}$, and $ma_z-\dot{\bar{M}}_{\dot{u}}$ are the added mass terms of the mass matrix.

The aerodynamic force terms can be expressed as functions of the stability derivatives of the motion variables involved. Thus, the aerodynamic force terms in Eq. (7) can be expressed as,

8 $$\begin{aligned} \begin{aligned}{}&D_a = D_e+\dot{\bar{D}}_uu+\dot{\bar{D}}_ww+\dot{\bar{D}}_qq\\&L_a = L_e+\dot{\bar{L}}_uu+\dot{\bar{L}}_ww+\dot{\bar{L}}_qq\\&M_a = M_e+\dot{\bar{M}}_uu+\dot{\bar{M}}_ww+\dot{\bar{M}}_qq \end{aligned} \end{aligned}$$

where $D_e$, $L_e$, and $M_e$ are the steady state values of the drag, lift, and moment coefficients, respectively, and $\dot{\bar{D}}_u$, $\dot{\bar{D}}_w$, $\dot{\bar{D}}_q$, $\dot{\bar{L}}_u$, $\dot{\bar{L}}_w$, $\dot{\bar{L}}_q$ $\dot{\bar{M}}_u$, $\dot{\bar{M}}_w$, and $\dot{\bar{M}}_q$ are the respective dimensional stability derivatives of drag, lift, and moment. The added mass coefficients in Eq. (7) and the stability derivatives needed for the aerodynamic part of the model in Eq. (8) are extracted using CFD-based methodology as explained in section “Stability derivative extraction”. The centre of gravity, centre of buoyancy, and inertia coefficients of the aerostat are obtained from the CAD model analysis.

The buoyancy and gravitational force terms in Eq. (7) can be expressed as,

9 $$\begin{aligned} \begin{aligned}{}&D_g = (mg-B)\sin {\alpha }\\&L_g = -(mg-B)\cos {\alpha }\\&M_g = -x_bB\cos {\alpha }-z_bB\sin {\alpha } \end{aligned} \end{aligned}$$

where *B* is the aerostat buoyancy and $x_b$ and $z_b$ are the coordinates of the aerostat centre of buoyancy.

Thus, the matrix representation of Eq. (7) can be given as in Eq. (10),

10 $$\begin{aligned} \begin{aligned}{}&\begin{bmatrix} m-\dot{\bar{D}}_{\dot{u}} &{}\quad 0 &{}\quad ma_z-\dot{\bar{D}}_{\dot{q}}\\ 0 &{}\quad m-\dot{\bar{L}}_{\dot{w}} &{}\quad ma_x+\dot{\bar{L}}_{\dot{q}}\\ ma_z-\dot{\bar{M}}_{\dot{u}} &{}\quad ma_x+\dot{\bar{M}}_{\dot{w}} &{}\quad I_y-\dot{\bar{M}}_{\dot{q}} \end{bmatrix} \begin{bmatrix} \dot{u}\\ \dot{w}\\ \dot{q} \end{bmatrix}= \begin{bmatrix} D_e\\ L_e\\ M_e \end{bmatrix} \\&\quad +\begin{bmatrix} \dot{\bar{D}}_u &{}\quad \dot{\bar{D}}_w &{}\quad \dot{\bar{D}}_q\\ \dot{\bar{L}}_u &{}\quad \dot{\bar{L}}_w &{}\quad \dot{\bar{L}}_q\\ \dot{\bar{M}}_u &{}\quad \dot{\bar{M}}_w &{}\quad \dot{\bar{M}}_q-ma_xU_e-ma_zW_e \end{bmatrix} \begin{bmatrix} u\\ w\\ q \end{bmatrix}+ \begin{bmatrix} D_T\\ L_T\\ M_T \end{bmatrix}+ \begin{bmatrix} (mg-B)\sin {\alpha }\\ {}-(mg-B)\cos {\alpha }\\ {}-x_bB\cos {\alpha }-z_bB \end{bmatrix} \end{aligned} \end{aligned}$$

The terms in the matrix, $$\begin{bmatrix}D_T~~L_T~~M_T\end{bmatrix}^T$$, of Eq. (10) represent the axial and vertical forces and the moment due to the tether. As the drag due to the tether segments is comparatively negligible compared with the aerostat drag, the tension forces alone are considered for the simulation. Resolving the tension of the tether will give the $D_T$, $L_T$, and $M_T$. The tether force components can be obtained based on the formulations presented in section “Tether model”.

### Stability derivative extraction

Aerodynamic stability derivatives associated with the mathematical model of the aerostat are extracted using a CFD-based methodology^21^. The proposed methodology involves a small-amplitude periodic oscillation of the aerostat model with respect to the corresponding directions for which stability derivatives are needed. In the current study, a longitudinal model is presented, and the oscillations are performed in the axial direction (surge), vertical direction (heave), and around the pitch axis.

**Figure 3:**
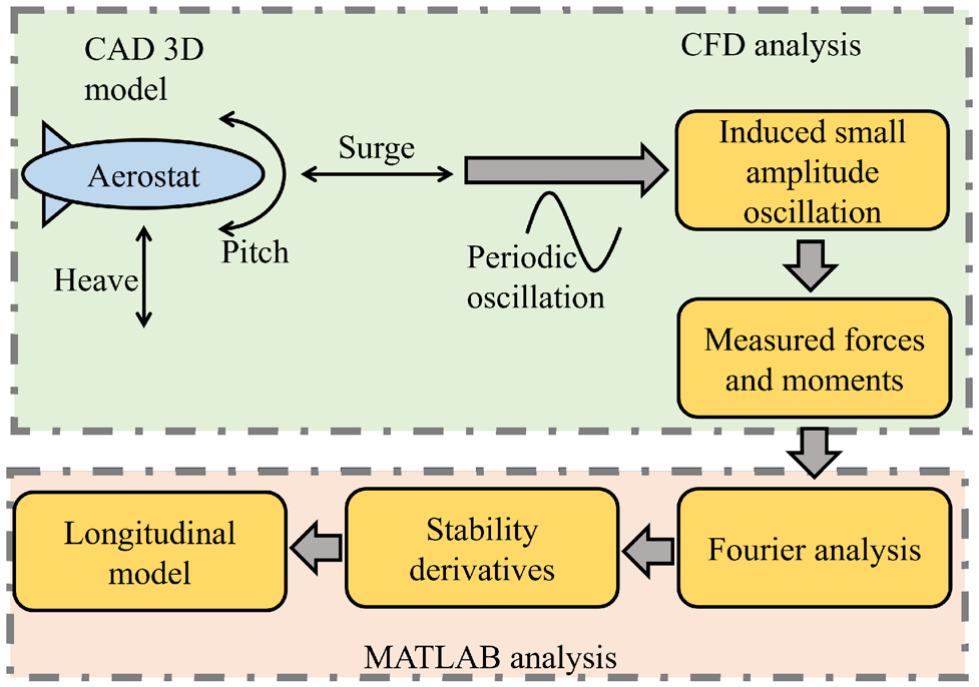
A schematic representation of the methodology adopted for the stability derivatives estimation.

The aerodynamic stability derivative calculation of the aerostat 3D model using CFD can be described using the schematic diagram shown in Fig. 3. A sinusoidal oscillation is induced in the aerostat model, and the response of the model is collected for multiple time periods of the oscillation. One complete cycle of response is analysed using Fourier analysis^21,22^.

The CFD computational setup, validation of the computational parameters used for the study, and validation of the aerostat 3-D model used for the analysis are available in the previous works of the authors^20–22^.

### Tethered aerostat model

The tether model and the aerostat model are coupled together by properly setting the geometrical constraints of the models. The attitude of the top node of the tether is provided as the confluence point of the aerostat. The resolved horizontal and vertical forces of the aerostat are applied to the top node of the tether.

**Figure 4:**
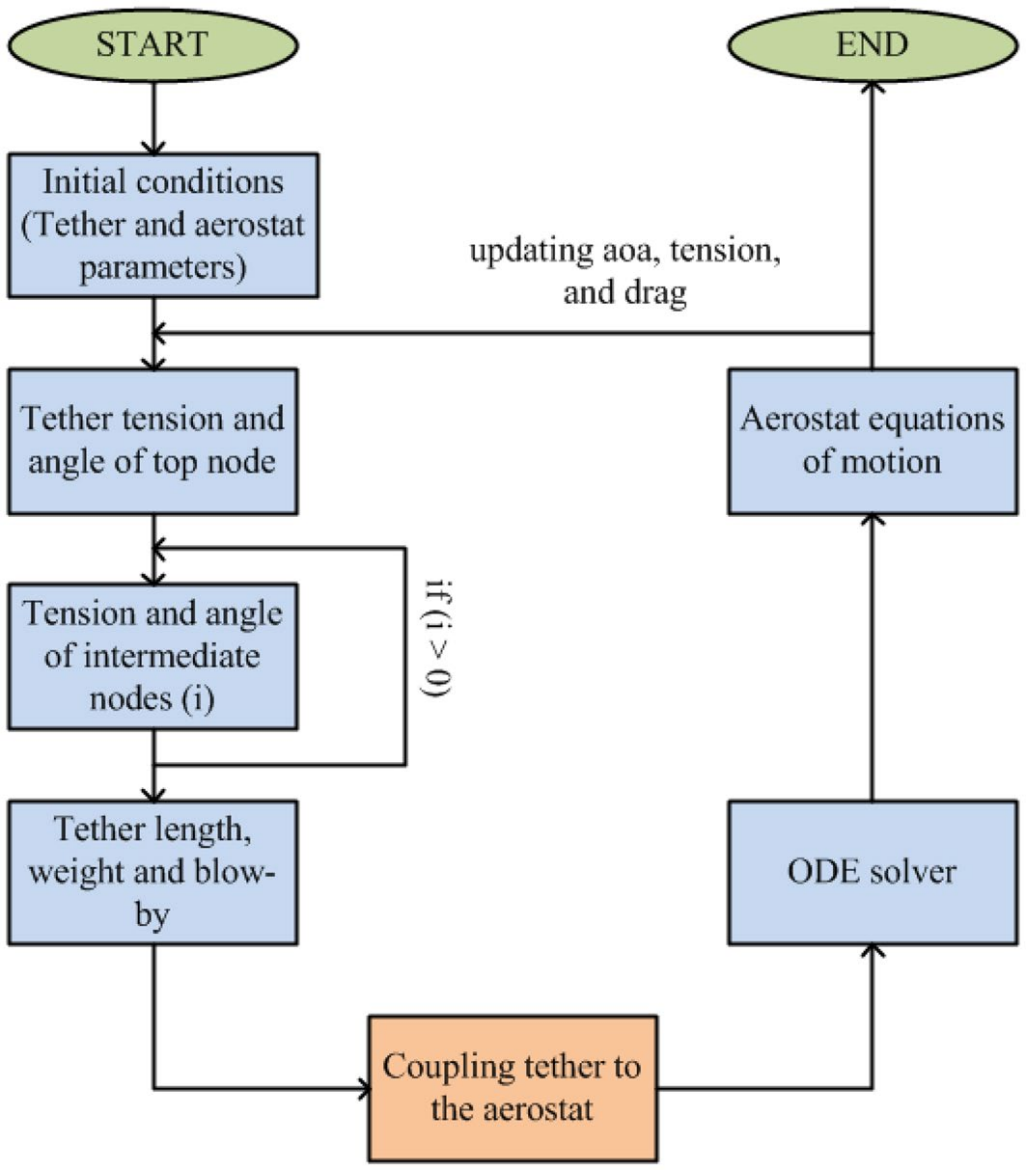
Simulation scheme adopted for the tethered aerostat simulation.

A flow chart explaining the complete scheme for the mathematical modelling of the tethered aerostat is shown in Fig. 4.

MATLAB 2020b is used for the simulation of the tethered aerostat model. The simulation is started by providing the needed geometrical parameters of the tether and aerostat. As the stability derivatives of the aerostat are obtained for a mean wind speed of 7.5 m/s, the tethered aerostat model is also simulated for the same velocity.

The simulation starts by calculating the tether tension and the tether angle of the top node of the tether by considering the steady-state conditions, and the equilibrium of forces can be given as specified in Eq. (11).

11 $$\begin{aligned} \begin{aligned}{}&T\sin {\theta }=B-W\\&T\cos {\theta } = D \end{aligned} \end{aligned}$$

The tension and angle of the following segments of the tether can be calculated using the equations specified in section “Tether model” in a recursive manner. As the tether segments are connected in series, this calculation can continue until the *n*th node is reached. Once the full tether profile is obtained, the total length of the tether, the weight of the tether, and the blow-by caused by the wind can be calculated from the *X* and *Z* coordinates of the tether segments. This procedure can be used to obtain the tether profile for various wind conditions. As the position coordinates of the top node of the tether can be obtained from the tether profile, the position coordinates of the aerostat need not be transformed to the earth-fixed reference frame. They can be directly obtained from the equations of motion of the aerostat and tether.

The non-stiff ordinary differential equations of the aerostat model were solved using the ODE45 numerical solver available with MATLAB 2020b. The simulation was conducted for 1000 s with a step size of 1 s and an initial condition of 100 m for altitude and 0 for all other states. As the drag calculation of the aerostat is needed for the tether model, the complete tethered aerostat model equations are considered in the ODE45 function. Thus, the drag and, thereby, the tether profile are updated for each iteration of the solver. The coupling of the tether model to the aerostat model is performed by providing the tension force from the tether to the aerostat and by specifying the initial position of the aerostat based on the top node position of the tether.

## Results and discussion

### Validation results

In this section, a set of results obtained from the validation analysis done as part of the current study is presented. Validation of the stability derivative extraction methodology and the aerostat tether model are discussed in the following sections.

#### Validation of the stability derivative extraction methodology

The methodology used to estimate or extract the longitudinal axis stability derivatives was validated using experimental data available in the open literature for a similar geometry^23^. The derivatives $\dot{L}_{\dot{w}}$ and $\dot{M}_{\dot{q}}$ were extracted by applying sinusoidal oscillations in their respective axes, and the obtained results are given in Table 1.

**Table 1:** Validation of the stability derivative extraction methodology.

Stability derivative	Current study	Theoretical value	Wang^23^
$\dot{L}_{\dot{w}}$	− 0.0457	− 0.0430	− 0.042
$\dot{M}_{\dot{q}}$	− 0.00339	− 0.0033	− 0.00332

#### Validation of the aerostat tether model

**Figure 5:**
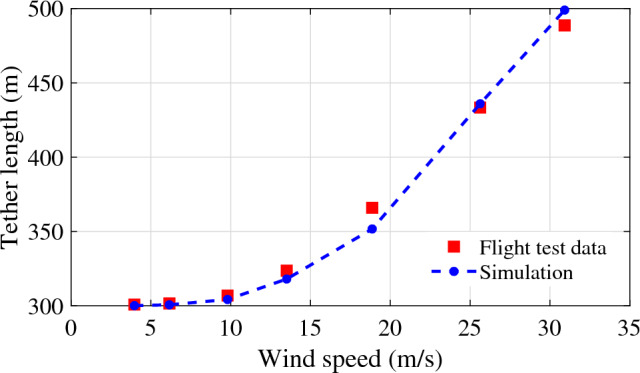
Validation of the presented tether model.

The tether model presented in section “Tether model” is validated using the flight test data of a 250 m^3^ aerostat^26^. The variation of the tether length for different wind amplitudes was compared, as shown in Fig. 5. The results show considerable similarity with the reported results and thus validate the presented tether model.

### Stability derivatives

The mathematical model of the tethered aerostat presented here was considered to be operating at an altitude of 100 m from ground level with a 10 degree AoA. In this section, the stability derivatives associated with the vehicle’s aerodynamic model are presented.

**Figure 6:**
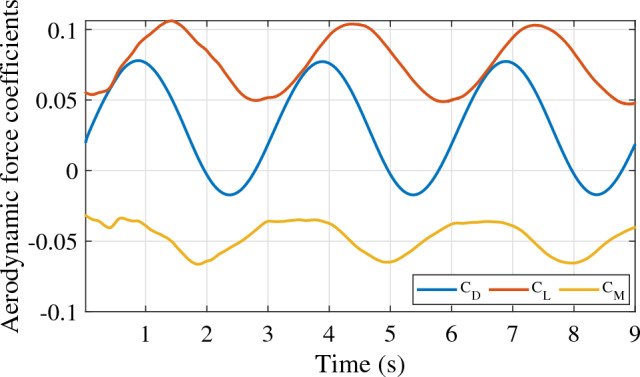
Aerostat force response for surge motion.

**Figure 7:**
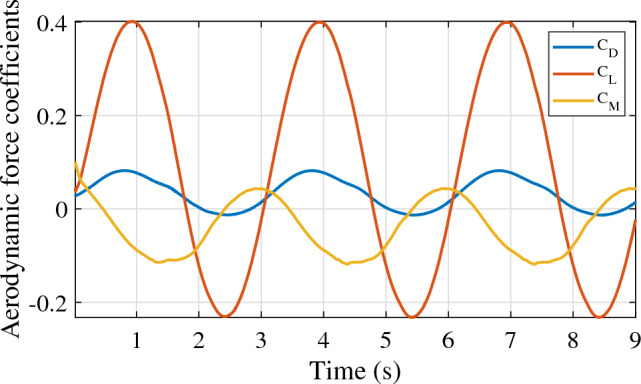
Aerostat force response for heave motion.

**Figure 8:**
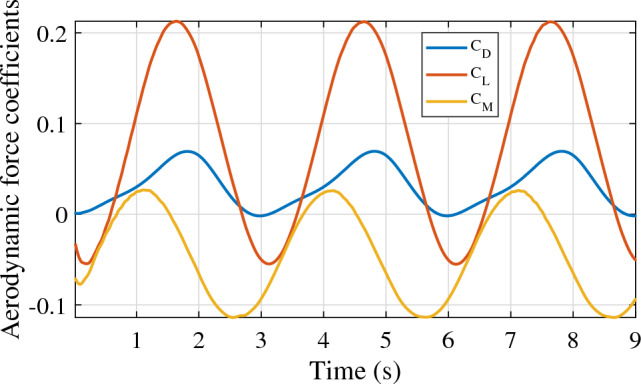
Aerostat force response for pitch motion.

As mentioned in section “Stability derivative extraction”, three motions are associated with the aerostat 3D model for stability derivative estimation. Figure 6 shows the response of the LTA vehicle undergoing surge oscillation with a 1 m/s amplitude and a time period of oscillation of 3 s. The non-dimensional lift, drag, and pitching moment were obtained for three consecutive cycles, as shown in the figure. The stability derivatives involved in the surge motion of the aerostat are given in Table 2. Similarly, the responses of the vehicle for heave and pitch oscillations are shown in Figs. 7 and 8, respectively. The stability derivatives associated with the heave and pitch oscillations of the aerostat are given in Tables 3 and 4, respectively. All the stability derivative values are expressed as non-dimensional quantities, as the force and moments were measured as non-dimensional coefficients, and time was non-dimensionalized by dividing it with the oscillation time period. For the aerostat model simulation, these derivatives were considered dimensional quantities, as all the mechanical parameters are dimensional. Table 5 gives the list of stability derivatives used for the analysis.

**Table 2:** Stability derivatives due to surge motion.

Derivative	Value
$\dot{D}_{\dot{u}}$	0.0193
$\dot{D}_{{u}}$	− 0.019
$\dot{L}_{\dot{u}}$	0.012
$\dot{L}_{{u}}$	− 0.0007
$\dot{M}_{\dot{u}}$	0.0017
$\dot{M}_{{u}}$	− 0.013

**Table 3:** Stability derivatives due to heave motion.

Derivative	Value
$\dot{D}_{\dot{w}}$	0.0196
$\dot{D}_{{w}}$	− 0.017
$\dot{L}_{\dot{w}}$	0.134
$\dot{L}_{{w}}$	− 0.11
$\dot{M}_{\dot{w}}$	0.0372
$\dot{M}_{{w}}$	− 0.0026

**Table 4:** Stability derivatives due to pitch motion.

Derivative	Value
$\dot{D}_{\dot{q}}$	0.00098
$\dot{D}_{{q}}$	− 0.008
$\dot{L}_{\dot{q}}$	0.005
$\dot{L}_{{q}}$	0.0015
$\dot{M}_{\dot{q}}$	0.0033
$\dot{M}_{{q}}$	0.00014

**Table 5:** Aerostat stability derivatives used for the simulation analysis.

Parameter	Description	Value
$\dot{\bar{M}}_w$	Moment derivative due to vertical velocity	− 11.3229
$\dot{\bar{M}}_q$	Moment derivative due to pitch velocity	− 0.6369
$\dot{\bar{D}}_{\dot{u}}$	Drag derivative due to axial acceleration	− 16.63477
$\dot{\bar{D}}_{\dot{q}}$	Drag derivative due to pitch acceleration	0.8501547
$\dot{\bar{L}}_{\dot{w}}$	Lift derivative due to vertical acceleration	− 115.9107
$\dot{\bar{L}}_{\dot{q}}$	Lift derivative due to pitch acceleration	− 4.476338
$\dot{\bar{M}}_{\dot{u}}$	Moment derivative due to axial acceleration	− 7.444886
$\dot{\bar{M}}_{\dot{w}}$	Moment derivative due to vertical acceleration	− 160.5588
$\dot{\bar{M}}_{\dot{q}}$	Moment derivative due to pitch acceleration	− 14.21907
$\dot{\bar{D}}_u$	Drag derivative due to axial velocity	− 16.9854
$\dot{\bar{D}}_w$	Drag derivative due to vertical velocity	− 15.3459
$\dot{\bar{D}}_q$	Drag derivative due to pitch velocity	− 6.96121
$\dot{\bar{L}}_u$	Lift derivative due to vertical velocity	− 0.65086
$\dot{\bar{L}}_w$	Lift derivative due to vertical velocity	− 95.4811
$\dot{\bar{L}}_q$	Lift derivative due to pitch velocity	1.305291
$\dot{\bar{M}}_u$	Moment derivative due to axial velocity	− 59.257

### Analysis of the tethered aerostat model

The simulation results of the tether and aerostat models are presented in this section. MATLAB 2020b is used for the analysis.

**Figure 9:**
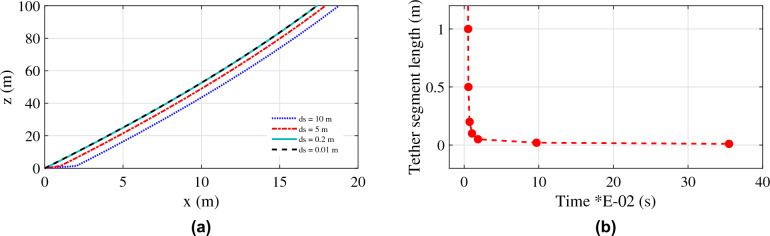
(**a**) Tether profile obtained for different tether segment lengths; and (**b**) computational time variation with the tether segment length.

The tether model considered in this research comprises a number of discrete segments, as shown in Fig. 1. The discretization of the tether considered for this research was performed after a set of studies that investigated the effect of the number of segments and the length of each segment on the tether profile. Different tether segment lengths were considered for the model, and the response of the tether profile was simulated for a wind with an amplitude of 12 m/s, as shown in Fig. 9a. For the longer segments, the model failed to accurately demonstrate the tether profile. As the segment length reduces to 1 m, the tether profile converges. Further reduction in the segment length does not show any further improvement. As the length of the tether segment decreases, the number of tether segments increases, which adds to the computational cost. A comparison of the simulation time for different segment lengths is shown in Fig. 9b. The computational cost increases as the number of segments increases. By considering a trade-off between accuracy and computational cost, the tether segment length is selected as 0.1 m for further analysis.

**Figure 10:**
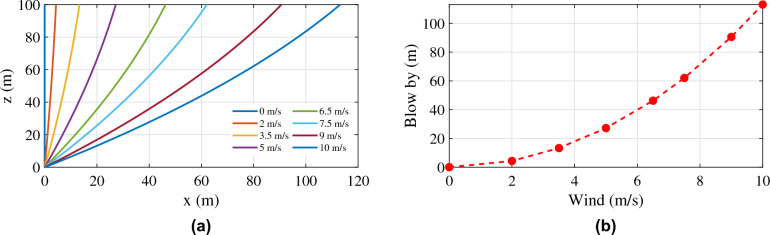
(**a**) Tether profile for different wind amplitudes with a fixed altitude of operation; and (**b**) blow-by with a fixed altitude of operation.

**Figure 11:**
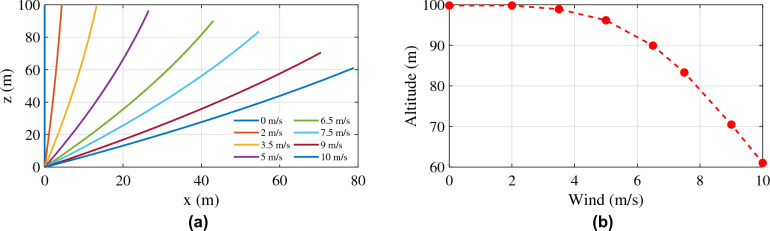
(**a**) Tether profile for different wind amplitudes with fixed tether length; and (**b**) dip in the altitude with a fixed length tether.

Even though the simulation studies deal with fixed wind conditions, the ability of the tether model to respond to different wind conditions has to be investigated. Here in this section, the tether profile was simulated for various wind amplitudes, and the results are shown in Figs. 10 and 11. Two conditions were considered: (i) the altitude of operation was kept constant by allowing the tether to vary its length, and (ii) the length of the tether was kept constant. Figure 10a shows the response of the tether to the first condition. This condition demands active control over the tether length based on the altitude of the top node and the tension on the tether, which is beyond the scope of this thesis. The tether should be released if the tension grows beyond a particular limit and the altitude is reduced, and the tether should be retracted if the tension is low and the altitude is increasing. Fig. 11a shows the response of the tether to the second condition. Since the length of the tether is fixed, the altitude of operation cannot be maintained at 100 m. This condition demands active control of the aerostat to maintain its altitude by opposing the motion due to wind, which is beyond the scope of this thesis. This will make the control system complex, and the payload capacity will decrease due to the additional control equipment on board. Figures 10b and 11b show the axial (blow-by) and vertical displacement of the top node of the tether for a fixed amplitude of operation and a fixed tether length operation. For the previous set of analyses, fixed weight, buoyancy, lift, and drag forces were considered to replace the aerostat forces. The altitude of the top node of the tether, the blow-by of the tether due to wind, and the total tether length for each wind condition may vary with the actual aerostat model incorporated into the tether model.

**Figure 12:**
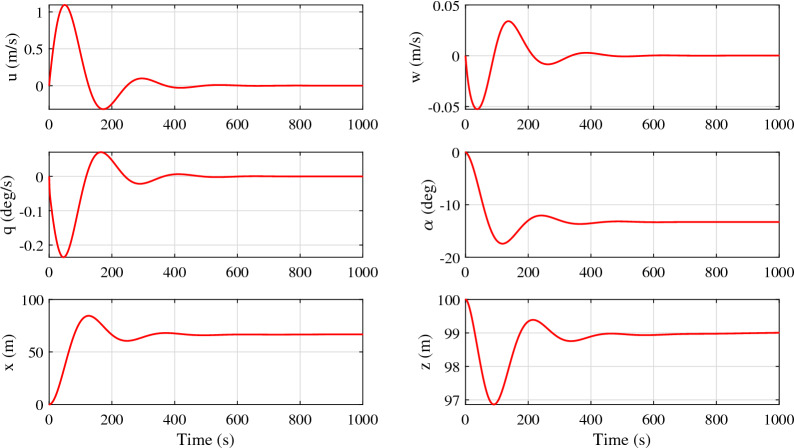
Response of the single-tethered aerostat undergoing a steady wind of 7.5 m/s.

The response of the tethered aerostat model for the mean wind velocity of 7.5 m/s is shown in Fig. 12. The aerostat came to rest after 500 s, as shown in the figure. The forward velocity of the aerostat settles to zero after a peak overshoot. The vertical velocity also reaches zero after a negative overshoot, as shown in the figure. The presented model is able to damp out the oscillations and allows the model to settle down in a stable condition, as shown in the figure.

**Figure 13:**
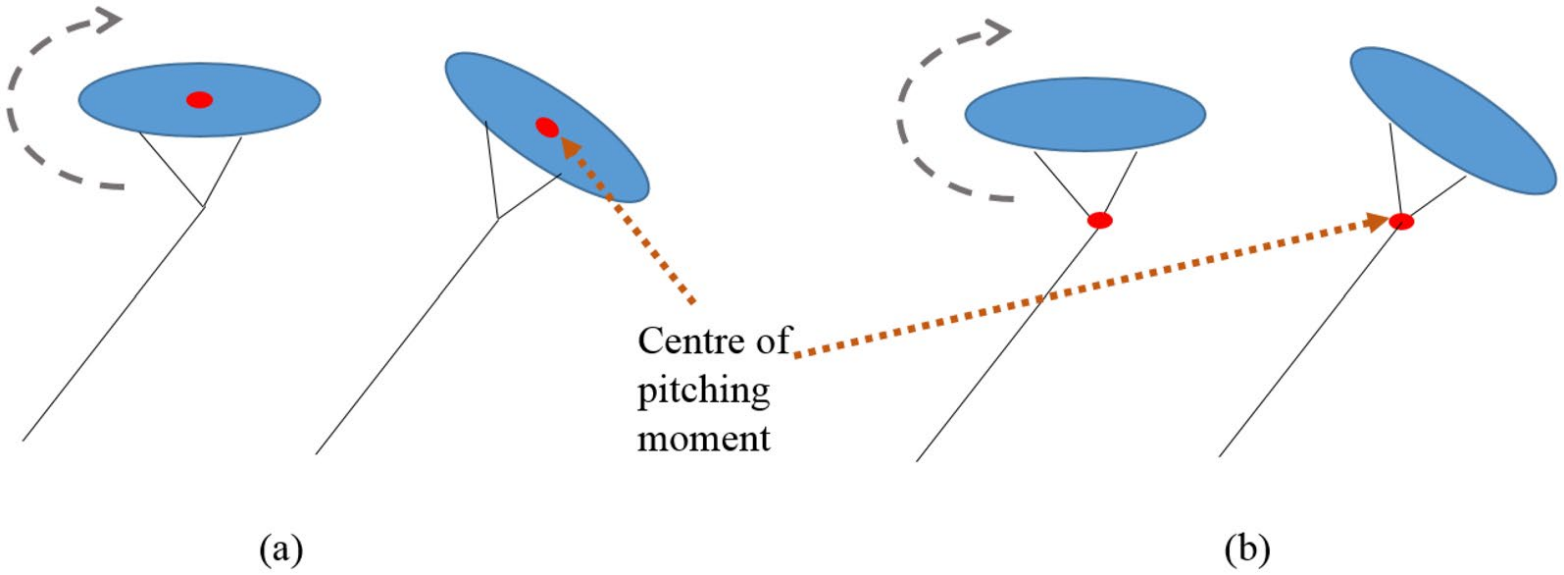
Schematic diagram showing the pitch centre of the tethered aerostat: (**a**) aerostat centre of volume as pitch centre; and (**b**) tether confluence point as pitch centre.

The pitch angle in the response is found to be negative, as shown in Fig. 12. This can be explained as the effect of the selection of the pitching centre for the aerostat while estimating the stability derivatives. The aerostat centre of volume was used as the pitching centre for the current analysis, as shown in Fig. 13a. As the leashes of the aerostat were assumed to be rigid along with the tether, the pitching of the aerostat has to be about the confluence point of the tether, as shown in Fig. 13b. This deviation in the pitching centre caused improper pitching of the aerostat and drove the aerostat to a negative pitch angle, as shown in Fig. 12.

The position of the aerostat can be obtained from the X and Z coordinates of the aerostat, as shown in Fig. 12. As the equations of motion were initiated with the position coordinates of the topmost node of the tether, the aerostat position can be obtained directly without any axis transformation. The altitude of operation was found to be slightly less than 100 m which shows the necessity of an active control system for the aerostat. In a similar manner, the blow-by caused by the wind drifted the aerostat up to 68 m from its initial position. An active control surface will help in controlling the blow-by of the aerostat.

**Figure 14:**
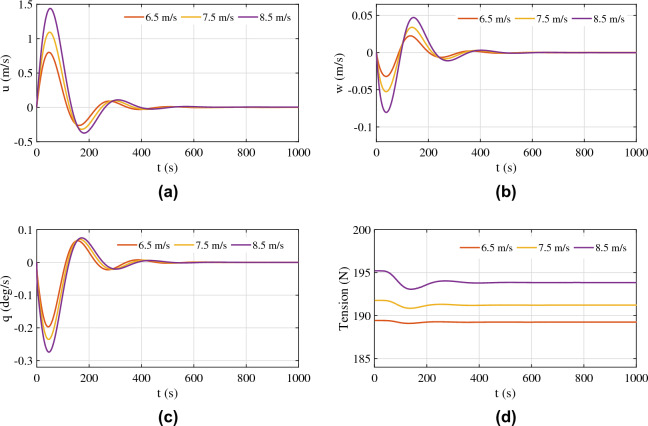
Response of the tethered aerostat for wind velocities close to the operating velocity: (**a**) forward velocity, (**b**) vertical velocity, (**c**) pitch rate, and (**d**) tether tension.

As mentioned previously, the presented model works in the vicinity of the mean wind velocity of 7.5 m/s. The responses of the tethered aerostat model for three different wind velocities are shown in Fig. 14.

As the wind velocity increases, the forward velocity of the aerostat increases with a hike in the overshoot, as shown in Fig. 14a. The time taken for the response to settle also increases with the wind. Similar behaviour is exhibited by the vertical velocity, as shown in Fig. 14b. The pitch angle shows considerable variation with the wind velocity. This behaviour once again suggests the need for a proper pitch control system for the aerostat. The negative pitch was the result of the selection of the aerostat centre of volume as the pitching moment centre for the estimation of the stability derivatives.

**Figure 15:**
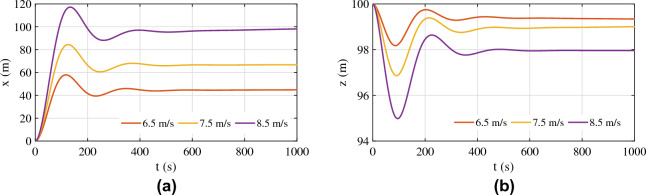
Response of the tethered aerostat for wind velocities close to the operating velocity: (**a**) horizontal position and (**b**) vertical position.

The tension force on the aerostat tether is shown in Fig. 14d. The magnitude of tension is lowest for the 6.5 m/s wind, and the maximum tension was obtained for the 8.5 m/s wind, which is the maximum wind velocity considered. As the wind velocity increases, the amplitude of the tension force on the tether shows a large initial value and then settles down to a lower amplitude. For lower wind velocities, this behaviour is not dominant. The time taken to settle the tension force response also increases with increasing wind velocity. The horizontal and vertical positions are shown in Fig. 15.

## Conclusion

An investigation of the longitudinally decoupled dynamic model of the single-tethered aerostat was carried out in this paper. The model parameters (stability derivatives and added mass terms) of the aerostat aerodynamic model estimated from the CFD-based analysis were investigated. To the best knowledge of the authors, the longitudinal mathematical modelling of an LTA vehicle is reported for the first time.

A two-dimensional model for the aerostat tether was developed. The presented model was validated using experimental data available for a tethered aerostat flight test. The tether length for different wind conditions was compared with the current simulation results for validation. The effect of segment length on the tether profile was analysed, and it was found that as the tether length decreases (the number of tether segments increases), the tether profile converges to a smooth profile. A longitudinally decoupled dynamic model of the aerostat was considered in the current study by assuming that the lateral dynamics were negated. The added mass terms and the stability derivatives of the aerostat were estimated using a CFD-based analysis. The model was found to be valid around the mean wind velocity with which the stability derivatives were estimated. The tether model was coupled to the aerostat model by initiating the aerostat with the tether tension, weight, and position of the top node of the tether. The MATLAB ODE45 numerical solver was used for solving the aerostat equations of motion along with the tether dynamics. The model was able to respond to different wind conditions as expected. The negative pitching of the aerostat was because the stability derivatives were investigated by considering the aerostat centre of volume as the pitching moment centre instead of the tether confluence point.

As a future direction, the lateral dynamics of the vehicle can be investigated based on the presented methodology. An investigation into the effect of confluence points on the stability derivatives of the vehicle is another open challenge in this direction. The investigation of proper control algorithms to stabilise the tethered aerostat and the efforts to make the mathematical model a generalised one by incorporating non-linear cable dynamics into the tether are also identified as open challenges for further research. An investigation into the scalability of the stability derivatives can also be done to generalise the methodology for a wide range of aerostats.

## Data Availability

All the data are available from the corresponding author and can be accessed via corresponding email after clearly stating the intention and permission to conduct research that requires our data.
